# The role of horizontal connections for the modulation of border-ownership selective neurons in visual cortex

**DOI:** 10.1186/1471-2202-16-S1-P176

**Published:** 2015-12-18

**Authors:** Nobuhiko Wagatsuma, Rudiger von der Heydt, Ernst Niebur

**Affiliations:** 1 School of Science and Engineering, Tokyo Denki University, Hatoyama, Hiki, Saitama, Japan; 2 Krieger Mind/Brain Institute, Johns Hopkins University, Baltimore, MD, USA

## 

**Figure 1:**
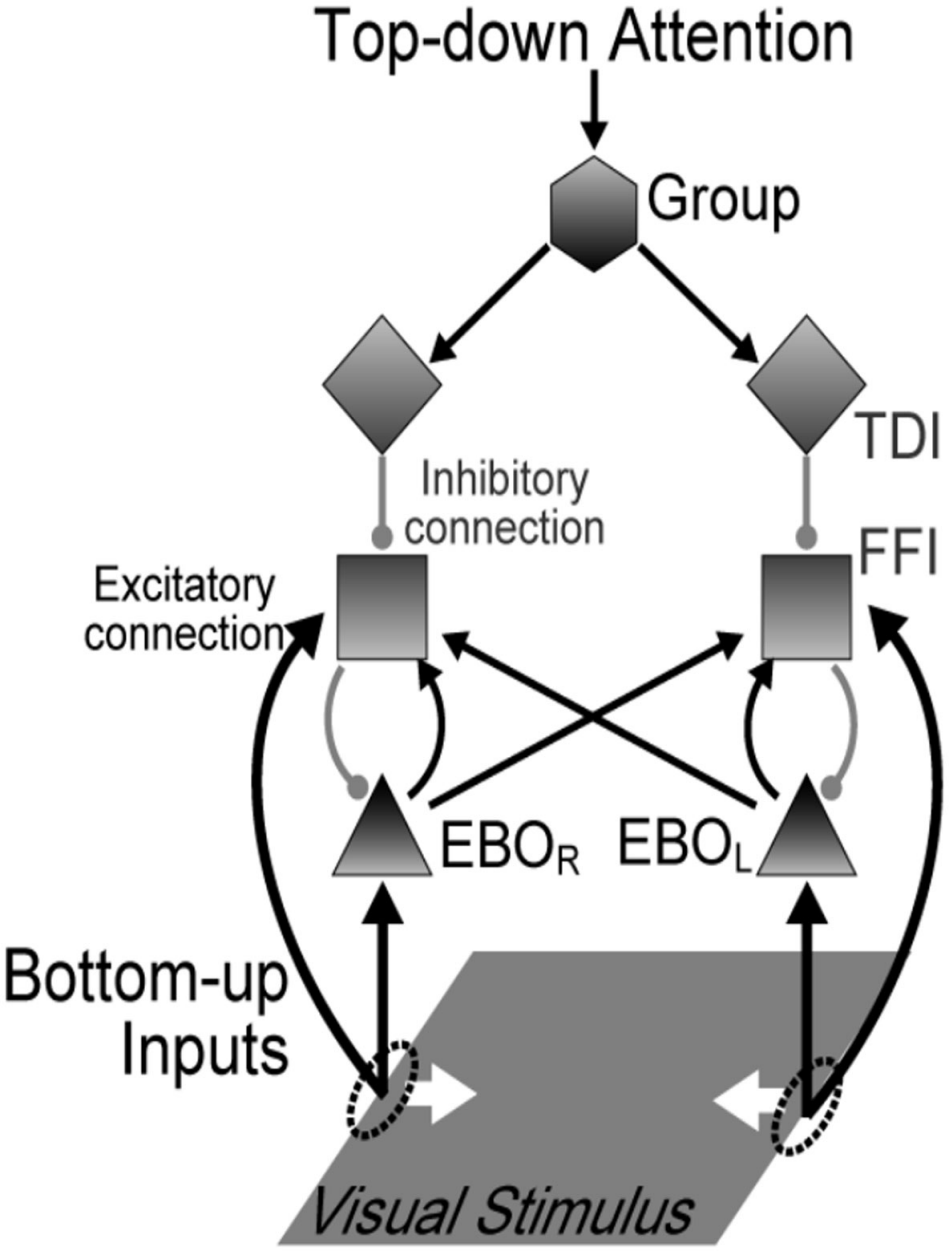
**Circuit of the dis-inhibition model**. The network was organized into 2 identical microcircuit units and consisted of 4 types of neurons [4]: excitatory BOS neurons (triangles, EBO), excitatory grouping neurons (hexagon, G), feedforward inhibitory neurons (squares, FFI) and top-down inhibitory neurons (diamonds, TDI). The activity of a G neuron increased when a consistent figure was present in the receptive fields of EBO neuron pairs projecting to it [5], and it increased further when selective attention was directed to the object represented by this G neuron [6]

Border-ownership selectivity (BOS) codes for the direction of a foreground object relative to the background [1]. Attention modulates the responses of BOS neurons in cortical areas V2, both for the firing rate [2] and in terms of spike synchrony which can occur over long cortical distances [3]. Here, we develop a network model of spiking neurons based on dis-inhibitory feedback [4]. Specifically, we consider the potential influence of intra-areal ("horizontal") connections for attention-induced modulation of BOS neurons in visual cortex. In our model (Figure 1), horizontal connections (oblique lines) connect excitatory BOS (EBO) neurons representing one part of a figure to feed-forward inhibitory (FFI) neurons representing another figure part. Since horizontal connections are weakly myelinated or non-myelinated, signal propagation along them is slow and interactions through them are subject to substantial delays, roughly proportional to the distance between the neurons they connect. We therefore investigated the influence of communication delays on spike-spike synchrony between BOS neurons. Our simulation results of the network with short delays are in agreement with experimental data, showing attentional enhancement of firing rates of EBO neurons and, at the same time, reduction of spike-spike synchrony. In contrast, simulations with more realistic (longer) delays could not reproduce experimental results. Our results suggest that horizontal connections in early cortical areas cannot explain the observed synchrony structure between BOS neurons and are unlikely to form the substrate of figure-ground segregation.
